# Field generated nematic microflows via backflow mechanism

**DOI:** 10.1038/s41598-020-57944-5

**Published:** 2020-01-29

**Authors:** Žiga Kos, Miha Ravnik

**Affiliations:** 1 University of Ljubljana, Faculty of Mathematics and Physics, Jadranska 19, 1000 Ljubljana, Slovenia; 2 Jožef Stefan Institute, Jamova 39, 1000 Ljubljana, Slovenia

**Keywords:** Fluids, Liquid crystals

## Abstract

Generation of flow is an important aspect in microfluidic applications and generally relies on external pumps or embedded moving mechanical parts which pose distinct limitations and protocols on the use of microfluidic systems. A possible approach to avoid moving mechanical parts is to generate flow by changing some selected property or structure of the fluid. In fluids with internal orientational order such as nematic liquid crystals, this process of flow generation is known as the backflow effect. In this article, we demonstrate the contact-free generation of microfluidic material flows in nematic fluids -including directed contact-free pumping- by external electric and optical fields based on the dynamic backflow coupling between nematic order and material flow. Using numerical modelling, we design efficient shaping and driving of the backflow-generated material flow using spatial profiles and time modulations of electric fields with oscillating amplitude, rotating electric fields and optical fields. Particularly, we demonstrate how such periodic external fields generate efficient net average nematic flows through a microfluidic channel, that avoid usual invariance under time-reversal limitations. We show that a laser beam with rotating linear polarization can create a vortex-like flow structure and can act as a local flow pump without moving mechanical parts. The work could be used for advanced microfluidic applications, possibly by creating custom microfluidic pathways without predefined channels based on the adaptivity of an optical set-up, with a far reaching unconventional idea to realize channel-less microfluidics.

## Introduction

Microfluidics manipulates fluids in confined geometries, typically channels, with dimensions of tens of micrometres, and has become a strong tool for analysis, manipulation, and detection^1–3^. Fluidic elements are developed to manipulate the fluid, such as pumps^4^, valves^5^, and membranes^6^. Together, typically combined on a microfluidic chip, these elements can perform various actions with the fluid, for example mixing^7^, transport^2^, and separation^8^. For manipulating the fluid, different physical mechanisms are demonstrated including electro-osmosis^9^, electro-wetting^10^, thermophoresis^11^, electrophoresis^12^, optical fields^13^, and acoustic waves^14^. Today, a major driver in microfluidics is to produce programmable and moving-part-free microfluidic networks^4,15,16^. However, the problem is that the established techniques produce fixed networks of channels, typically by pre-determined photolithographic templates^1^ or patterned surfaces^17^. Alternatively, only recently, new technologies allowing for a localized and channel-free manipulation of fluid are emerging, for example a chemically powered micropumps^18^ and microfluidics with fluid walls^19^.

Microfluidic functionality can be further expanded by using fluids with internal structure, such as for example nematic liquid crystals, where transport of colloidal cargo^20^, electric field switching of channel resistivity^21^, fabrication of microresonators^22^, manipulation of colloidal particles by groovy interfaces^23,24^, and generation of intertwined field structures^25^ have been demonstrated utilizing nematic orientational order and high responsiveness to external fields. Liquid crystals can form complex orientational structures^26^, which are then strongly coupled to the material flow^27^ and can lead to flow-induced structural transitions^28^ and also activity-driven microfluidics^29^. In a nematic microchannel, the effective fluid resistance is dependent on the orientation profile (director field) of the nematic molecules. This property has been used to induce a net flow by applying a periodically oscillating pressure difference in a channel^30^. However, this process works only if applied pressure is sufficiently large that velocity field induces enough distortion in the director profile. Another possibility to drive nematic flows is to utilize the mechanism of backflow. Backflow indicates the flow generation due to director reconfiguration. Backflow is demonstrated to importantly affect the dynamics in various dynamical processes, such as (i) the motion of topological defects in the defect coarsening dynamics^31–33^, (ii) the manipulation and anisotropic aggregation of colloidal particles^34,35^, (iii) the Non-Stokesian dynamics of colloidal particles^36^, and (iv) flow-enhancement and power requirement in pulsatile flows^37^. Backflow effects have been successfully implemented in driving flows of smectic liquid crystals^38–40^. Moreover, backflow is elementary in all liquid crystal display application as it causes flickering of display pixels upon switching (optical bounce), and needs to be engineered to minimize the effects^41^. The basic idea behind the backflow generated flows is to use external fields, such as electric or optical fields, to drive the distortions of the nematic director and generate the desired flow profiles.

The phenomena of flow generation is present also in other systems, such as swimming of microorganisms, which is known to require swimming strokes that are non-reciprocal in time^42^. Performing the same motion of flagella back and forth might generate a momentary motion of a microorganism, but in a long scale it remains still. Microswimmers have adapted to this by using non-reciprocal swimming strokes, caused for example by beating flagella or rotating helices^43^. Similarly, generation of nematic backflow is dependent on the reciprocity of the time modulation of director field that induced by the varying external fields. Slow reciprocal changes of the electric field — for example turning it slowly on and off — will typically result in backflow-generated flow profiles with opposite direction as electric field is slowly turned on and off, eventually leading to zero average flow in the sample. A possible solution to generate a net flow in nematic confinement is to use fast-varying external fields that lead to non-equilibrium director configurations. Such beating motion of the director field induced by oscillating electric fields was shown to create an average backflow profile, used to drive colloidal particles or net flows in smectic^38–40^ or nematic^34,35,44^ liquid crystals. Periodically modulated voltages are also used to drive the motion of Skyrmion nematic structures^45^. In the right regime of voltage modulation or confinement, such non-reciprocal transformation of chiral nematic field could be possibly also accompanied by strong backflow currents. Another possibility for the creation of average nematic flows is to generate rotating director structures, as for example by using rotating electric or optical fields. Both of the above mechanisms lead to non-reciprocal director motion in time and can generate an average backflow profile in a nematic sample.

In this article, we demonstrate the use of electric and optical fields to continually generate nematic flow profiles via backflow mechanisms. We use homogeneous electric field with square-like voltage modulation to drive nematic flows in planar and hybrid aligned (HAN) nematic cells, inducing an average net flow in the later case. We observe that the efficiency of flow-driving is increased if electric field with rotating polarization is used. This is further explored by using laser beams with rotating linear polarization, leading to vortex flow structures in homeotropic nematic cells. Vortex formation can be used in nematic channels to generate a net flow in left or right direction. This work is a step towards expanding the functionality of electric fields and in particular laser beams for creation of nematic flows, allowing for possible applications in microfluidics and manipulation of nematic colloidal or orientational structures. Utilization of such local flow generation could eliminate the need for external pressure pumps or pumps with moving parts and enable microfluidic setups with closed channels. Furthermore, the results of this article are a step towards channel-free microfluidics, where streamlines of nematic flows could be created by careful positioning of laser beams across the nematic sample with minimal contribution of the confinement effects.

## Results

The functionality of nematic materials is strongly related to their structure^20–22^. The dynamics of non-equilibrium nematic fluids is dependent on the temperature, pressure, and — in the context of this work — on the application of external fields, which cause effective elastic deformations in the molecular ordering^46^. Specifically, in this work we focus on electric and optical fields, as two capable and also experimentally viable contact-free flow-driving mechanisms. The results are obtained through numerical and analytical efforts, using mesoscopic continuum nematodynamic modelling based on the order parameter tensor which is solved by the hybrid lattice Boltzmann method^47,48^. The used mesoscopic approach is especially capable of fully accounting for the backflow effects. In the simulations, positive dielectric anisotropy of the nematic material was used. By changing the electric field magnitude or orientation, the nematic profile dynamics generates flow via backflow effect. For full explanation of theory, methods and used material parameters please see Theory and Methods section.

### Oscillating magnitude of homogeneous electric field in microchannels

**Figure 1:**
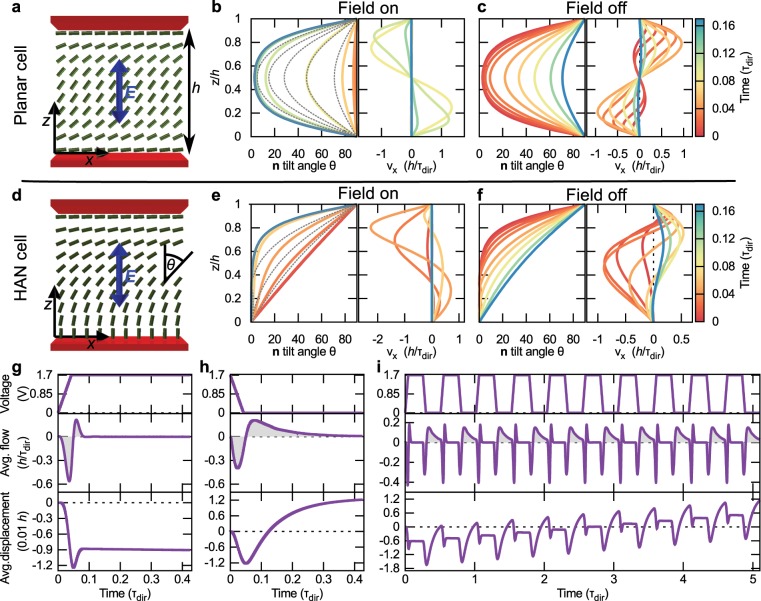
Microflow generation by homogeneous oscillating electric fields in simple nematic cells. (**a**) A schematic of a planar cell with a vertically applied electric field. Director tilt angle and the velocity profile are shown as the electric field is turned on (**b**) and off (**c**). In both cases, the fluid motion is bidirectional. The maximum displacement of the nematic molecules is  ≈ 0.06 *h*. The dotted line for the director tilt angle in (**b**) shows the director profile when the electric field is switched off. There is a mismatch between the profiles, which leads to a slight difference in the maximum displacement of the bidirectional motion. The displacement for turning the electric field off is larger for  ≈ 0.002 *h*. (**d**) A schematic of the director field and the applied electric field for a HAN cell. (**e**,**f**) Director and velocity profiles for turning the electric field on and off, respectively. During the switching process, there is a momentary average net flow in either left or right direction. (**g**) Applied voltage between top and bottom plate, average flow and average displacement for turning the electric field on. The averaging is performed across the whole cell height. After a long enough time an average displacement of  ≈ 0.009 *h* towards the left is established. (**h**) Measurement is repeated for turning the electric field off. A displacement of  ≈ 0.012 *h* towards the right is observed. (**i**) By applying a square-like voltage, we established a net average flow through a HAN cell. From the increasing average displacement in time, an average flow of 〈*v*〉_*z*,*t*_ = 2.4 ⋅ 10^−3^ *h*/*τ*_dir_ is calculated.

Figure 1a–c shows the director profile and the velocity profile in a nematic cell with planar anchoring at the top and the bottom surface after the electric field has been turned on and off. Turning the vertical electric field on in such planar nematic cell shows the properties of a Freedericksz transition. Homogeneous in-plane nematic orientation is unstable under the vertical electric field; however, the rate of director rotation is initially small as a large angle between the electric field and the director leads to a small torque acting on the nematic molecules. As the director turns for a moderate angle the transition is accelerated leading to a fast reconfiguration to the equilibrium profile. Due to the symmetry of a planar cell, the velocity profile is bidirectional — the velocity field in the top half of the cell is a inverse mirror image of the velocity field in the bottom half. Turning the electric field off generates again a bidirectional velocity profile which is generally in the opposite direction compared to the on switch. A maximum local displacement of the fluid is  ≈ 0.06 *h*, where *h* is the height of the cell (*h* is used as a unit of length throughout the article). Due to non-equilibrium nature of the transition, the director profile during the on switch is not exactly equal to the director profile due to the off switch, which leads to a difference of the total fluid displacement between the off and on switch of the electric field. Indeed, displacement is for  ≈ 0.002 *h* larger for switching the electric field off.

The symmetry of a planar cell in Fig. 1a–c in principle prevents any net flows in the sample. This symmetry requirement is broken in a HAN cell, consisting of a surface with a homeotropic (perpendicular) director orientation and a surface with planar orientation (Fig. 1d). Again, we have tested the response of the nematic director and the generated flow profile to the turning off and on of the vertical electric field. Alternatively, also a horizontal electric field or materials with negative dielectric anisotropy could be used to generate flow in an analogous manner. As shown in Fig. 1e,f, the shape of the director field and velocity profile is extensively different for the off and for the on switch of the electric field. For example, velocity maximum for the on switch is in the upper half of the cell, whereas for the off switch flow maximum is in the bottom half. Fig. 1g,h shows average flow (averaged over the cell thickness) and its time integral — i.e. average displacement. Nematic adapts rather quickly to the strong electric field — flow generation is negligible after *t* = 0.1 *τ*_dir_ (*τ*_dir_ is the director elastic relaxation time; for more see Theory and Methods). When the electric field is turned off, nematic reconfiguration is driven by slower elastic effects and flow is generated for a longer period of time. On switch of the electric field generates an average displacement of 0.009 *h* towards the left and off switch generates an average displacement of 0.012 *h* towards the right. We utilized the difference between left and right average displacements by applying a square wave-like voltage to the HAN cell (Fig. 1i) to generate time and thickness-averaged flow field of 〈*v*〉_*z*,*t*_ = 2.4 ⋅ 10^−3^ *h*/*τ*_dir_ in a HAN cell.

### Rotating homogeneous electric field in microchannels

**Figure 2:**
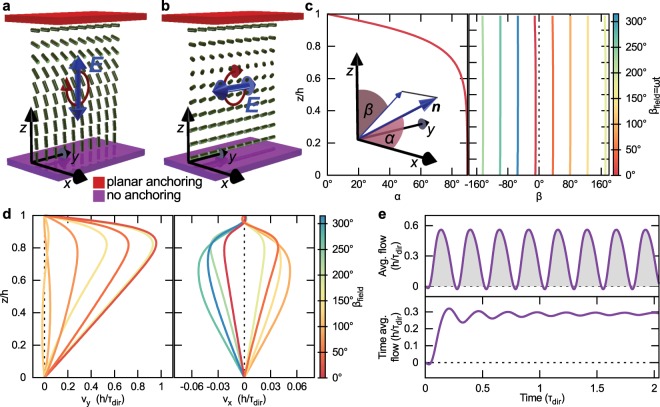
Flow generation by rotating homogeneous electric field. (**a**,**b**) Schematics of the director field and the electric field orientation at angles *β*_field_ = 0^°^ and *β*_field_ = 90^°^, respectively. The top plate imposes a planar anchoring along *x* direction and the bottom plate imposes no anchoring on nematic molecules. Electric field is rotated around  − *x* axis. (**c**) Director field tilt angle towards the *x* axis *α* and azimuthal angle in the *yz* plane *β* at different times (i.e. at different orientations of the applied electric field). *β*(*β*_field_) is continuous in time, but we plot it only for discrete values of *β*_field_. (**d**) Velocity field profile along the *y* and along the *x* direction during one rotation of the electric field. The period of oscillation of *v*_*y*_ is only 180^°^ in *β*_field_. On average, flow in the *x* direction cancels itself out, but there is a finite contribution along the *y* axis. (**d**) *v*_*y*_ averaged over the height of the cell (top row) and its time average (bottom row) revealing a total average flow of 0.3 *h*/*τ*_dir_.

The efficiency of flow generation by electric field profiles can be improved compared to Fig. 1 if rotating electric fields are used. Small average flow rate in HAN cells with oscillating voltage is a consequence of: (i) bidirectional flow along the cell thickness (Fig. 1e,f), (ii) in time, flow averaged over the cell thickness changes direction when the director field is adapting to voltage being turned on or off (Fig. 1g,h), and (iii) average fluid displacement for on and off voltage switch are in opposite directions. The idea behind using rotating electric fields is to generate a director distortion that is non-reciprocal in time — in line with the discussion of the symmetry of the Ericksen-Leslie equations in the Theory and Methods section — and could potentially lead to flow profiles that are predominantly in a single direction. We demonstrate a possible application of a rotating electric field in a nematic cell with a top surface with planar anchoring and a bottom surface with no anchoring of nematic molecules (see Fig. 2a,b).

The director field in Fig. 2 can be expressed in terms if the *x*, *y*, and *z* components as ${\boldsymbol{n}}=(\sin \alpha ,\sin \beta \cos \alpha ,$ $\cos \beta \cos \alpha )$ and the electric field rotates in the plane that is perpendicular to anchoring direction at the top surface ${\boldsymbol{E}}={E}_{0}\left(0,\sin {\beta }_{{\rm{field}}},\cos {\beta }_{{\rm{field}}}\right)$, where *β*_field_ = *ωt* + *π*/2, *E*_0_ = 1.7 V/*h*, and $\omega =12.3\ {\tau }_{\,{\rm{dir}}\,}^{-1}$ is the rotation frequency. The plane of the electric field combined with the nematic in-plane and no-anchoring boundary conditions is chosen in a way to promote continuous smooth director rotation without creation of defects. For example, using bottom plate with strong surface anchoring would lead to effective ’tearing’ of the continuous nematic director field and formation of topological defects, which might be interesting on its own, but from the perspective of realising continuous and smooth flow fields is likely undesirable. Figure 2a,b shows the director profile for *β*_field_ = 0^°^ and *β*_field_ = 90^°^, respectively. In (a) the director shows splay and bend deformation between the bottom and the top surface, while in (b) the director field is deformed purely in the twist profile. In Fig. 2c the director field is given in terms of *α* and *β* angles. The dependence of *α* on the vertical position *z* is the same at all times. This is a consequence of equal splay, twist, and bend elastic constants used in simulations. If the values of the elastic constants would not be equal, the *α*(*z*) profile would change in time. Angle *β* is found to be approximately homogeneous along the cell thickness, showing a delay of about 10^°^ behind the electric field orientation. Figure 2d shows the velocity profile in the simulated nematic cell under the application of a rotating electric field. In the *y* direction, flow is the largest at *β*_field_ ≈ 0^°^. *v*_*y*_ has a periodicity of 180^°^ rotation in *β*_field_. In the *x* direction, flow profile changes its sign as the electric field rotates for 180^°^, leading on average to zero net flow along the *x* axis. Figure 2e shows the flow averaged over the cell thickness. Average flow is mostly positive, except for a short period when the electric field is horizontal (*β*_field_ is 90^°^ or 270^°^). At longer times, a time-averaged flow of 0.3 *h*/*τ*_dir_ is established.

The backflow effect of a rotating director field in confined nematics can be under several approximations illustrated by a simple analytical calculation of a nematic liquid crystal confined between two plates at *z* = 0 and *z* = *h*, similar to geometrical set-up in Fig. 2a,b. Within the analytical consideration, the top plate is taken to impose a director orientation along the *x* axis and the bottom plate is assumed to impose a rotating director ${\boldsymbol{n}}=(0,\cos \omega t,-\sin \omega t)$ with the rotation frequency *ω* that is slow enough for the director in the bulk to be in an equilibrium configuration, which is given by ∇^2^***n*** = 0. Under these assumptions, the time-dependent director across the nematic cell can be written as:

1 $${\boldsymbol{n}}=\left(\sin \frac{\pi z}{2h},\cos (\omega t)\cos \frac{\pi z}{2h},-\sin (\omega t)\cos \frac{\pi z}{2h}\right),$$

where *h* is the cell thickness. Rotation of the director field in time generates a force on the nematic material, which can be calculated as a divergence of the viscous stress tensor (Eq. 11). In the *y* direction the force equals

2 $${f}_{y}=\frac{\pi \omega }{2h}\left[-{\alpha }_{2}{\sin }^{2}(\omega t)+{\alpha }_{3}{\cos }^{2}(\omega t)\right]\sin \frac{\pi z}{h},$$

where *α*_2_ and *α*_3_ are Leslie viscosity coefficients. There is a non-zero time average force after a large number of oscillations

3 $$\langle \,{f}_{y}\rangle =\frac{\pi \omega }{4h}{\gamma }_{1}\,\sin \frac{\pi z}{h}.$$

**Figure 3:**
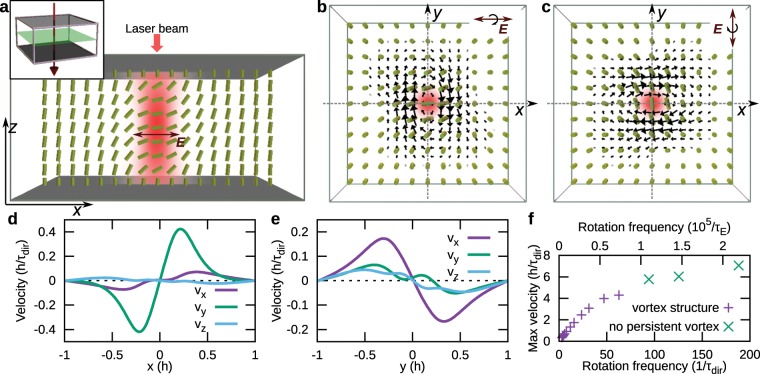
Vortex flow created by laser beam with rotating polarization. (**a**) Electric field intensity of the laser beam (red colormap) for polarization in the *x* direction and the corresponding director beam profile (green rods). Inset shows the set-up with beam direction (red arrow) and the the green plane at which the cross-sections of the field profiles are drawn in (**b**,**c**). The polarization of the laser beam is rotated around the  − *z* axis. The waist radius of the beam is taken to be 0.15 *h* and the Rayleigh length is 0.38 *h*. (**b**) Field structures when beam polarization is along the *x* axis, and (**c**) along the *y* axis. Black arrows show the velocity profile, which is of a vortex-like structure. (**d**,**e**) Vortex-like structure is confirmed by velocity cross-sections along (**d**) *x* direction and (**e**) *y* direction taken from panel (b). (**f**) Maximum velocity field increases with increasing frequency (shown in units of electric field time scale *τ*_E_ and director relaxation time scale *τ*_dir_), starts saturating, until at even larger frequencies (now shown), the flow would start to decrease and eventually become zero. In the "no persistent vortex regime" of frequency range, director field no longer follows the rotation of the electric field; however, director oscillations still generate strong velocity fields, but with no persistent vortex structure.

**Figure 4:**
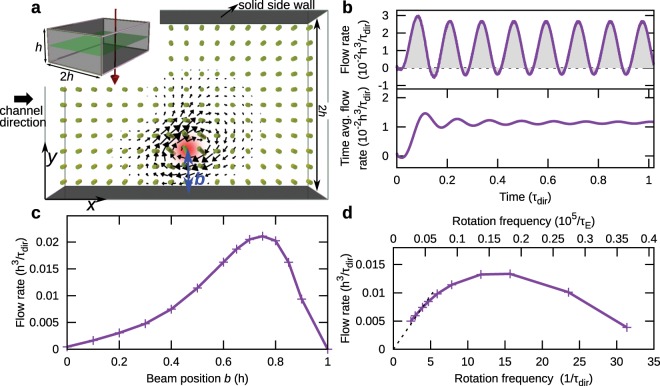
Net flow generated by laser beam with rotating polarization in proximity of side wall. (**a**) If the center of the laser beam is close to the side wall of a microfluidic channel, which imposes non-slip boundary condition, part of the vortex is suppressed. Inset shows the beam direction (red arrow) and the plane at which the cross-section of the relevant fields is drawn (in green). (**b**) Oscillation of the flow rate through the channel and its time average. Net flow rate in the channel is along the *x* direction. (**c**) Optimization of the beam center position in the channel. Too close to the boundary, the director field is strongly conditioned by the surface anchoring and does not follow the electric field. If the laser beam is centered in the middle of the channel, flow field is bidirectional with zero net flow. (**d**) Optimization of the flow rate with respect to rotation frequency (in units of electric field time scale *τ*_E_ and director relaxation time scale *τ*_dir_). At low frequencies the director field follows the electric field, but the flow magnitude is small, because of slow rotation of the director field. Linear response to the rotation frequency is shown by the dotted line. At higher frequencies, net flow generation is diminished. In given geometry, an optimal rotation frequency is  ≈ 14/*τ*_dir_.

Here, *γ*_1_ = *α*_3_ − *α*_2_. The calculated force (in *y* direction) in turn causes material flow in same direction. Equation 3 represents a solution to a minimal analytical model that explains how induced rotations of the director field can generate flow in a nematic cell, which is a result that is used in Figs. 2–4. Since typically in nematics $|{\alpha }_{2}| >  > |{\alpha }_{3}|$, flow maximum is generated at approximately $\sin \omega t=\pm 1$, which is also clearly observed in the numerical simulation (Fig. 2d,e).

### Flow fields induced by laser beams with rotating polarization

Optical beams could be used as an interesting source of rotating/modulating electric fields to drive flows in nematics. Here, we demonstrate, how a Gaussian beam with a rotating direction of linear polarization (i.e. not circular polarization) induces a vortex structure in homeotropic nematic cells (see Fig. 3). We model a Gaussian laser beam by taking the standard electric filed intensity profile determined analytically in para-axial approximation within optically isotropic media, which is a well known approach for considering the coupling between optical fields and nematic order^49^. Also, the relative magnetic permeability of the nematic is set to one, since coupling with the electric field is typically much stronger. The nematic deformation in homeotropic cells driven by a laser beam with rotating polarization is similar to the director deformation in the case of rotating homogeneous electric field in Fig. 2. The director deforms from a planar orientation in the center of the beam to a homeotropic orientation far away from the beam that is dictated by the boundary conditions on the confining surfaces. The deformation is continuous in time, generating a vortex-like structure in the *xy* plane, as shown in Fig. 3b,c. Velocity profiles (Fig. 3d,e) reveal that the vortex is not strictly two-dimensional and rotationally symmetric, which is a consequence of the periodic boundary conditions, complex flow generation where the driving forces do not form a cylindrically symmetric structure, and anisotropic viscosity of nematic liquid crystals. We also tested the frequency dependence of the maximum velocity magnitude generated using Gaussian beams with rotating polarization (Fig. 3f), which increases with the rotation frequency.

At large rotation frequencies (approximately above 75/*τ*_dir_), the director field can no longer follow the rotation of the electric field. In such fast regime, the director field shows back-and-forth rotations with high velocity amplitude (Fig. 3f); however, no clear persistent vortex structure is established. At even larger rotation frequencies than used in our simulations, the time-varying electric field would effectively start to behave as if static, because the nematic director reorientation would become to slow to respond at all to the electric field.

### Laser beams with rotating polarization for net flow generation in microchannels

Laser beams with rotating polarization can be also used to generate directional flows in nematic channels, which is demonstrated in Fig. 4 for rectangular channels with anchoring along the vertical direction at all four channel walls. If the laser beam spot is positioned close to one of the surfaces, the part of the vortex in the material flow that is closer to the surface gets dissipated more, leading to a net flow in the channel. Figure 4b shows the flow rate (i.e. volumetric flow rate through the channel) and the time-averaged flow rate in such configuration over time. The results are similar to Fig. 3, showing net flow primarily in one direction, leading to a substantial average flow in a channel. We probed the dependence of the average flow rate on the position of the beam (Fig. 4c) and the rotation frequency (Fig. 4d). If the beam is positioned at the center of the channel, symmetry prevents any net flow rate. If the beam is too close to the surface, surface anchoring suppresses the optical field-generated distortion of the director field, leading again to zero net flow rate. The optimal position of the laser bean center in our numerical set-up is  ≈ 0.75 *h* away from the side-wall. For small rotation frequencies, linear dependence of the flow rate on the frequency is expected and also observed, as the time scale of the rotation of the polarization is so small that the nematic is effectively in the local equilibrium. For fast rotation frequencies the net flow in the channel is decreased. This can be attributed to on average more vertical direction of the director field due to fast rotation of polarisation and anchoring conditions, and to narrower vortex structure which causes smaller difference in dissipation of both sides of the vortex flow. Figure 4 shows the optimal rotation frequency for beam position at *b* = 0.5 *h*. Rotating laser beam in Fig. 4 can act as a local flow pump. For a long channel, the viscous drag would limit the pumping efficiency, in which case more laser beams or a combination with other pumping methods would be required.

## Discussion

In this work, we demonstrate mechanisms of flow generation by electric or optical fields using backflow effects in nematic liquid crystals. The first approach for flow generation that we explore is abrupt switching of a homogeneous electric field in planar and hybrid aligned nematic cells. In a planar cell, such switching mechanism generates a bidirectional flow profile (Fig. 1a–c), which was already demonstrated to be effective in manipulation of colloidal particles in nematics^34^. In a hybrid aligned cell, square wave-like voltage modulation leads to an average flow in single direction (Fig. 1d–i). However, the effectiveness of flow generation by oscillating electric fields is reduced by the fact that during one period of voltage oscillation, most of the fluid flow generated during on-switching is back-compensated by counter-flow generated during off-switching, effectively generating only small net residual flow. We suggest flow generation using rotating electric fields or optical fields with rotating polarization. Such fields induce a nematic deformation that is non-reciprocal in time at any frequency of rotation and leads to mostly unidirectional flow in the case of homogeneous rotating electric field (Fig. 2). We demonstrate how a laser beam with rotating polarization in a homeotropic nematic cell generates a vortex-like flow structure around the centre of the beam (Fig. 3), which notably — as an alternative route — could be also achieved by a combination of electrodes or magnets. Provided that the nematic is confined in a microfluidic channel with no-slip walls, part of the vortex can get viscously dissipated at the side wall and a net flow is generated (Fig. 4). Such generation of nematic flow can be tuned by using the appropriate rotational frequency of the polarization. At low frequencies the time derivatives of the director field are small, generating small backflows. At too high frequencies the director response can no longer follow the changes in the optical field, leading to a diminished driving efficiency. This article explores flow generation mechanisms by smooth director field deformations, but there could be other routes to achive efficient flow generation relative to the given energy input. A possible new direction of research would be flow generation by creation and annihilation of topological defects within the nematic orientational field.

The magnitude of the flow fields in the simulations of field-generated microflows can be expressed in full dimension units and rescaled to channel dimensions typical for experiments. In Figs. 2 and 3, the dimensionless number characterizing the flow strength — i.e. Ericksen number — is moderate at $\,{\rm{Er}}\,=\frac{vh}{\Gamma L}\approx 0.5$, where *v* is a typical velocity magnitude in simulations, Γ the rotational viscosity parameter, and *L* the elastic constant (see Theory and Methods). The simulated nematic is in principle characterized by an intrinsic nematic lengthscale (nematic correlation length *ξ*_N_); however, since in the presented simulations of field-generated microflows the director deformations are continuous (i.e. without defects present), the system size is scalable, with the dynamics dependent primarily on the director field (and not the nematic correlation scale, which is the scale for variations of the degree of order). Such consideration allows to estimate the flow magnitude in selected cell geometries. For the same Ericksen number $\,{\rm{Er}}\,=\frac{{v}_{0}{h}_{0}}{\Gamma L}$ and for the same material parameters *L* and Γ, the typical velocity *v* in a cell of thickness *h* scales depending on the simulation values *v*_0_ and *h*_0_ approximately as $v={v}_{0}\frac{{h}_{0}}{h}$. For a typical cell thickness in experiments *h* = 10 μm and typical parameter values of *L* = 4.8 pN and Γ = 15 (Pas)^−1^ such scaling estimate gives a typical maximum velocity of *v* = 6 μm/s, which is comparable to flow magnitudes in experiments with nematic flows^34^. Note also that the time average local magnitude of the bidirectional flow in ref. ^34^ was less than 1% of the maximum flow amplitude, while in Fig. 2 of this article the average flow through the entire channel is about 30% of the maximum local flow magnitude.

The presented research explores the use of laser beam profiles to generate local flows in nematic cells. Laser beams are already extensively used (in nematics) as tweezers to entrap, manipulate, and assemble colloidal particles^50^. The proposed mechanism would extend the functionality of laser beams as an experimental tool for flow generation in nematic liquids. Experimentally, rotating polarization could be achieved by circularly polarized laser beam and a rotating polarizer. Typically, nematic ordering is observed between crossed polarizers. In principle, polarization microscopy technique does not necessary exclude laser beams with rotating polarization, as the viewing light and the driving beam can be applied at different wavelength regimes. In simulations, we assume that nematic remains at a constant temperature. In experiments, temperature variation would depend on the absorption and heat diffusion properties of the nematic and the surrounding material. Increased temperature would decrease the nematic degree of order (or even melt the nematic to an isotropic phase) and magnitude of the generated flow. Alternatively, colloidal object can be moved also by strong laser beams that locally melt the nematic^51^. Using laser beams to drive nematic flows is a step towards channel free and moving parts free microfluidics, where continuous deformation of fluid’s internal structure acts as a local generator of flow. The possibility of local fluid pumps could enable closed microfluidic circuits, which can be beneficial in terms of the lower use of the material or simply repeated microfluidic actions on the same fluid. Furthermore, using multiple laser beams with possibly opposite direction of polarization rotation, one could create flow networks without channels. Since microfluidic channels are typically imprinted using lithography techniques, channel networks have to be prepared in advance. Contrary, using an optical field, flow structure could be adapted and changed during experiment without actually constructing microfluidic chips, but rather by a fully reconfigurable chip platform that could be arbitrarily programmed to perform functions and actions with the fluid. This would require an optical set-up, but a nematic cell could retain a minimalistic geometry. Finally, using rotating laser beams can provide a microfluidic functionality with no predefined channels, where flow networks and flow magnitude are shaped using optical fields without external pumps or moving parts, which could be further designed to create custom flow pathways.

## Theory and Methods

### Modelling of nematic flows

We performed numerical simulations of nematodynamics using an approach with a nematic tensorial order parameter **Q**. The scalar degree of order *S* and the main ordering axis (director) ***n*** are the largest eigenvalue and the corresponding eigenvector of the Q-tensor, respectively. Equilibrium configuration of a nematic structure corresponds to a minimum of the free energy, written in the Landau-de Gennes form as^46^:

4 $$F={\int }_{V}\left\{\frac{A}{2}{Q}_{ij}{Q}_{ji}+\frac{B}{3}{Q}_{ij}{Q}_{jk}{Q}_{ki}+\frac{C}{4}{({Q}_{ij}{Q}_{ji})}^{2}+\frac{L}{2}\frac{\partial {Q}_{ij}}{\partial {x}_{k}}\frac{\partial {Q}_{ij}}{\partial {x}_{k}}-\frac{1}{2}{\epsilon }_{0}\left(\bar{\epsilon }{E}_{i}^{2}+\frac{2}{3}{\epsilon }_{\,{\rm{a}}}^{{\rm{mol}}\,}{Q}_{ij}{E}_{i}{E}_{j}\right)\right\}{\rm{d}}V,$$

where *A*, *B*, *C* are material phase parameters, *L* is the elastic constant, *ϵ*_0_ vacuum permittivity, $\bar{\epsilon }$ average permittivity for a nematic, and ${\epsilon }_{\,{\rm{a}}}^{{\rm{mol}}\,}$ molecular dielectric anisotropy. Summation over repeated indices is assumed. Time evolution of the tensor order parameter is described by the Beris-Edwards equation^52^:

5 $${\dot{Q}}_{ij}-{S}_{ij}=\Gamma {H}_{ij},$$

where Γ is the rotational viscosity parameter, *H*_*ij*_ is the molecular field driving the system towards equilibrium

6 $${H}_{ij}=-\,\frac{1}{2}\left(\frac{\delta F}{\delta {Q}_{ij}}+\frac{\delta F}{\delta {Q}_{ji}}\right)+\frac{1}{3}\frac{\delta F}{\delta {Q}_{kk}}{\delta }_{ij},$$

and *S*_*ij*_ is the generalized advection term, dictating the response of nematic molecules in shear flow:

7 $${S}_{ij}=(\zeta {A}_{ik}-{\Omega }_{ik})\left({Q}_{kj}+\frac{{\delta }_{kj}}{3}\right)+\left({Q}_{ik}+\frac{{\delta }_{ik}}{3}\right)(\zeta {A}_{kj}+{\Omega }_{kj})-2\zeta \left({Q}_{ij}+\frac{{\delta }_{ij}}{3}\right){Q}_{kl}\frac{\partial {v}_{k}}{\partial {x}_{l}},$$

where *A*_*ij*_ is symmetric part of the velocity gradient tensor *A*_*ij*_ = (∂_*i*_*v*_*j*_ + ∂_*j*_*v*_*i*_)/2 and Ω_*ij*_ the antisymmetric part Ω_*ij*_ = (∂_*i*_*v*_*j*_ − ∂_*j*_***v***_*i*_)/2, ***v*** is the fluid velocity, and *ζ* the alignment parameter. Material flow is described by the generalized Navier Stokes equation and the incompressibility condition

8 $$\rho \left[\frac{\partial {v}_{i}}{\partial t}+\left({v}_{j}{\partial }_{j}\right){v}_{i}\right]={\partial }_{j}{\sigma }_{ij},$$

9 $$\nabla \cdot {\boldsymbol{v}}=0,$$

respectively, where *ρ* is the fluid density and ***σ*** is the stress tensor, which for nematics takes the form:

10 $$\begin{array}{ll}{\sigma }_{ij}\,= & -\frac{\delta F}{\delta {\partial }_{j}{Q}_{kl}}{\partial }_{i}{Q}_{kl}-({p}_{0}-f){\delta }_{ij}-\zeta {H}_{ik}\left({Q}_{kj}+\frac{{\delta }_{kj}}{3}\right)-\zeta \left({Q}_{ik}+\frac{{\delta }_{ik}}{3}\right){H}_{kj}+2\zeta \left({Q}_{ij}+\frac{{\delta }_{ij}}{3}\right){Q}_{kl}{H}_{kl}\\  & +\,{Q}_{ik}{H}_{kj}-{H}_{ik}{Q}_{kj}+2\eta {A}_{ij}+{\epsilon }_{0}{E}_{i}\left(\bar{\epsilon }{E}_{j}+\frac{2}{3}{\epsilon }_{\,{\rm{a}}}^{{\rm{mol}}\,}{Q}_{jk}{E}_{k}\right).\end{array}$$

*p*_0_ is an external pressure contribution, *f* the free energy density from Eq. 4, and *η* the isotropic viscosity parameter. Equation for the stress tensor captures the transmission of forces through the bulk, anisotropic viscosity of nematics, generation of backflow currents, and coupling to the electric field^53^.

Model equations for nematodynamics are solved using a hybrid lattice Boltzmann approach, which includes a finite difference method for Q-tensor time evolution (Eq. 5) and lattice Boltzmann method for fluid flow (Eqs. 8, 9)^47,48^. At the interfaces, no-slip boundary condition and fixed strong anchoring of nematic molecules are used. At the ends of the channels, periodic boundary conditions are used. The numerical results are expressed in the units of cell height *h* and director relaxation time scale *τ*_dir_ = *h*^2^/(Γ*L*). In all the simulation, the height of the simulation box is *h* = 146 *ξ*_N_, expressed with the nematic correlation length ${\xi }_{{\rm{N}}}=\sqrt{L/(A+B{S}_{{\rm{eq}}}+\frac{9}{2}C{S}_{{\rm{eq}}\,}^{2})}$. The following values of other model parameters are used: *ζ* = 0.94, *η* = 1/Γ, *B*/*A* = 12.3, and *C*/*A* = 10.1. The strength of the electric field can be expressed by considering a typical time-scale for the nematic to adapt to the applied field ${\tau }_{E}=1/(\Gamma {\epsilon }_{0}{\epsilon }_{\,{\rm{a}}}^{{\rm{mol}}\,}{E}^{2})$. The ratio between the director relaxation time scale and the electric field time scale in simulations with homogeneous electric field is *τ*_dir_/*τ*_*E*_ ≈ 200. This would correspond to a material with *L* = 4.8 pN, ${\epsilon }_{\,{\rm{a}}}^{{\rm{mol}}\,}=35$ and applied voltage of 1.7 V between the top and the bottom plate (as used in Fig. 1). Optical fields are modelled through an effective electric field of Gaussian shape^49^. The maximum magnitude of the effective electric field within the beam is 2.5-times higher as in Figs. 1 and 2.

### Symmetry of Ericksen-Leslie equations

Ericksen-Leslie theory is a director-based description of nematic hydrodynamics^46^. Within the theory, the stress tensor ${\sigma }_{ij}={\sigma }_{ij}^{\,{\rm{v}}{\rm{i}}{\rm{s}}{\rm{c}}{\rm{o}}{\rm{u}}{\rm{s}}}+{\sigma }_{ij}^{{\rm{E}}{\rm{r}}}+{\sigma }_{ij}^{{\rm{M}}{\rm{a}}{\rm{x}}{\rm{w}}{\rm{e}}{\rm{l}}{\rm{l}}\,}-{p}_{0}{\delta }_{ij}$ is given as a sum of viscous stress tensor, Ericksen stress tensor and Maxwell stress tensor. Individual contributions are expressed as:

11 $${\sigma }_{ij}^{\,{\rm{viscous}}\,}={\alpha }_{1}{n}_{i}{n}_{j}{n}_{k}{n}_{l}{A}_{kl}+{\alpha }_{2}{n}_{j}{N}_{i}+{\alpha }_{3}{n}_{i}{N}_{j}+{\alpha }_{4}{A}_{ij}+{\alpha }_{5}{n}_{j}{n}_{k}{A}_{ik}+{\alpha }_{6}{n}_{i}{n}_{k}{A}_{jk},$$

12 $${\sigma }_{ij}^{\,{\rm{Er}}}=-\frac{\delta F}{\delta {\partial }_{j}{n}_{k}}{\partial }_{i}{n}_{k}+{f}_{{\rm{Frank}}},$$

13 $${\sigma }_{ij}^{\,{\rm{Maxwell}}\,}={E}_{i}{D}_{j}-\frac{1}{2}{\epsilon }_{0}{\epsilon }_{kl}{E}_{k}{E}_{l}{\delta }_{ij},$$

where *A*_*ij*_ is the symmetric velocity gradient tensor, ${N}_{i}={\dot{n}}_{i}-{\epsilon }_{ijk}{\omega }_{j}{n}_{k}$ the corotational time derivative of the director, ${\omega }_{i}=\frac{1}{2}{\epsilon }_{ijk}{\partial }_{j}{v}_{k}$, *α*_1−6_ the Leslie coefficients, *f*_Frank_ elastic free energy in the Frank formulation, ***D*** electric displacement field, and **ϵ** the dielectric tensor. Magnetic field effects are omitted from the above formulation of the Maxwell stress tensor. Most of the experiments on nematic microfluidics are performed at small Reynolds numbers. Eliminating the inertial terms and in the absence of any external forces, Navier Stokes equation can be reduced to the Stokes equation

14 $${\partial }_{j}{\sigma }_{ij}=0.$$

The divergence of the Ericksen and Maxwell stress tensor can be further simplified

15 $${\partial }_{j}\left({\sigma }_{ij}^{\,{\rm{Er}}}+{\sigma }_{ij}^{{\rm{Maxwell}}\,}\right)=-\,{h}_{k}{\partial }_{i}{n}_{k}=-\left({\gamma }_{1}{N}_{k}+{\gamma }_{2}{A}_{kl}{n}_{l}\right){\partial }_{i}{n}_{k},$$

where *h*_*k*_ = *γ*_1_*N*_*k*_ + *γ*_2_*A*_*kl*_*n*_*l*_ is the molecular field, *γ*_1_ = *α*_3_ − *α*_2_, and *γ*_2_ = *α*_2_ + *α*_3_. We see that there is a symmetry on time reversal in Stokes equation with the nematic stress tensor. If ***v***(***r***) is the solution to the Stokes equation at given director field ***n***(***r***) and its time derivative $\dot{{\boldsymbol{n}}}({\boldsymbol{r}})$, then upon reversing the time also gives a solution to the Stokes equation. If the profile of the periodically oscillating director field in time is reciprocal under time reversal, backflow effect will generate flow fields that for longer times average out to zero. Such director field distortion can be created by slowly oscillating external fields with nematic orientation always corresponding to a minimum of the free energy. To avoid this and to induce average local flow, we use two approaches to create backflow-generating director profiles that are non-reciprocal in time: (i) The rate at which the electric field is switched on or off is much shorter then the relaxation time of nematic director, thus creating non-equilibrium director field profiles that differ for switching the electric field on or off, (ii) rotating electric field (or polarization of light) create director distortion that is non-reciprocal in time even for small frequencies of rotation. Other approaches might include for example annihilation (and creation) of defects or Freedericksz transitions.
